# Singing more, singing harsher: occurrence of nonlinear phenomena in a primate’ song

**DOI:** 10.1007/s10071-023-01809-7

**Published:** 2023-07-17

**Authors:** Walter Cristiano, Teresa Raimondi, Daria Valente, Chiara De Gregorio, Valeria Torti, Valeria Ferrario, Filippo Carugati, Longondraza Miaretsoa, Laura Mancini, Marco Gamba, Cristina Giacoma

**Affiliations:** 1 Department of Life Sciences and Systems Biology, University of Turin, 10123 Turin, Italy; 2 Groupe d’Étude et de Recherche sur les Primates de Madagascar (GERP), II M 78 BIS Antsakaviro, B.P 779 Antananarivo, Madagascar; 3 Ecosystems and Health Unit, Environment and Health Department, Italian National Institute of Health, 00161 Rome, Italy

**Keywords:** Nonlinearities, Vocal folds, Phonation, Vocal fatigue, Singing primates, *Indri indri*

## Abstract

Nonlinear phenomena (NLP) in animal vocalizations arise from irregularities in the oscillation of the vocal folds. Various non-mutually exclusive hypotheses have been put forward to explain the occurrence of NLP, from adaptive to physiological ones. Non-human primates often display NLP in their vocalizations, yet the communicative role of these features, if any, is still unclear. We here investigate the occurrence of NLP in the song of a singing primate, the indri (*Indri indri*), testing for the effect of sex, age, season, and duration of the vocal display on their emission. Our results show that NLP occurrence in indri depends on phonation, i.e., the cumulative duration of all the units emitted by an individual, and that NLP have higher probability to be emitted in the later stages of the song, probably due to the fatigue indris may experience while singing. Furthermore, NLP happen earlier in the vocal display of adult females than in that of the adult males, and this is probably due to the fact that fatigue occurs earlier in the former because of a greater contribution within the song. Our findings suggest, therefore, that indris may be subjected to physiological constraints during the singing process which may impair the production of harmonic sounds. However, indris may still benefit from emitting NLP by strengthening the loudness of their signals for better advertising their presence to the neighboring conspecific groups.

**Supplementary Information:**

The online version contains supplementary material available at 10.1007/s10071-023-01809-7.

## Introduction

Nonlinear phenomena (NLP) are produced by irregular vibrations of the vocal folds (Herzel et al. 1995). They represent any abruption in the synchronization of the vocal folds leading to unstable oscillations (Tyson et al. 2007) and resulting in a variety of perceptually harsh and rough vocalizations (Fitch et al. 2002). NLP generally occurring in mammalian emissions belong to the following four major groups: (1) chaotic phenomena arising from aperiodic oscillations (Fitch et al. 2002), (2) splitting of harmonic cycles due to differential vibration of the vocal folds (Wilden et al. 1998; Riede et al. 2004), (3) simultaneous emission of sounds produced by different phonatory sources within the vocal tract (Reby et al. 2016), and (4) sudden changes in the fundamental frequency (Riede et al. 2004).

Previous research provided various explanations for the occurrence of NLP in animal vocalizations. A broad of evidence shows that the emergence of nonlinearities in mammalian vocalizations may reflect the emotional state of the emitter, e.g., stress or arousal (Blumstein and Récapet 2009; Blesdoe and Blumstein 2014; Fuchs et al. 2021; Marx et al. 2021; Massenet et al. 2022). Such sounds are indeed often associated with aversive states, such as rage, pain, and fear in animals, including humans (Anikin 2020; Marx et al. 2021). Alternative hypotheses highlighted that NLP may serve the function of grabbing the attention of conspecifics in the context of alarm calls in the presence of predators (Townsend and Manser 2011; Karp et al. 2014; Volodin et al. 2018), or triggering the listener’s attention by adding unpredictability within calling and thus make these signals hard to ignore (Fitch et al. 2002; Townsend and Manser 2011; Karp et al. 2014). Previous studies also suggested that NLP can facilitate the transmission of indexical cues to conspecifics, including individual identity, and that they can be exploited thanks to natural selection (Fitch et al. 2002). Besides adaptive explanations, other evidence indicates that nonlinear vocal dynamics may be a mere by-product of physiological constraints involving a certain degree of vocal fatigue (Riede et al. 2004; Boucher 2008; Krajewski et al. 2010; Weissmann et al. 2020; Sportelli et al. 2022). As such, fatigue may reflect a short-term effort necessary for dealing with challenging vocal performances (Weissmann et al. 2020) or a long-term one due to constant intense activity over time, for example during a season (Vannoni and McElligott 2009). In this framework, it has been argued that NLP may be associated with individual characteristics of the emitter such as body size, health, reproductive state, age, or sex (Riede 2007; Vannoni and McElligott 2009; Cazau et al. 2016; Weissmann et al. 2020; Anikin et al. 2021; Marx et al. 2021). NLP may therefore play also a role in sexual selection by conveying information on male quality to females, as previously reported in baboons, deer, and whales (Fischer et al. 2004; Reby and Charlton 2012; Cazau et al. 2016). However, in organized communicative systems, nonlinear vocal signals may be critical in providing information about individuality and urgency without complex neural control (Fitch et al. 2002).

Non-human primates have been indicated as animals that frequently exhibit NLP in their vocalizations (Fitch et al. 2002). Recent findings show that it is the peculiar morphology of their larynx that makes them susceptible to nonlinearities and that the prevention of such features in humans seems to be favored by a loss of complexity in the vocal anatomy. This, in turn, may have contributed to the development of speech under cognitive control (Nishimura et al. 2022). Additionally, the avoidance of nonlinearities in humans seems to elicit an attraction for clearly harmonic sounds and a preference for consonance rather than dissonance perception (Wagner et al. 2020). However, beyond humans, there are other primate species capable of emitting harmonic signals, such as singing primates (De Gregorio et al. 2022a). It is, therefore, of particular interest to investigate the occurrence of NLP in these species to unravel the significance of such unique features.

Among singing primates, there is only one lemur species, the indri (*Indri indri*). Indris inhabit the mountain rainforests of Madagascar, live in small family groups including a reproductive pair and its offspring (Torti et al. 2017), and they occupy territories that are defended through the emission of songs (Torti et al. 2013), which spread through the forest for kilometers (Spezie et al. 2022). Indri songs represent long calls that require a high energy demand (Zanoli et al. 2020) similar to what happens in the songs of other singing primates (De Gregorio et al. 2022a). We refer to songs to indicate a series of different types of frequency-modulated notes, i.e., units, uttered according to a hierarchical structure (De Gregorio et al. 2022a). Indris emit sexually dimorphic songs showing major differences in both frequency and temporal features (Giacoma et al. 2010; Gamba et al. 2016; Zanoli et al. 2020) starting with harsh *roars* given by all group members, followed by harmonic frequency-modulated units organized in phrases (Zanoli et al. 2020). Youngsters and subadults may sing with parents, in duets or choruses (Giacoma et al. 2010), and undergo changes during growth according to their age and sex (De Gregorio et al. 2021). Furthermore, parent–offspring turn-taking dynamics affect the vocal behavior of the adults (De Gregorio et al. 2022b). Female songs are less stereotyped than male songs (Zanoli et al. 2020), and both probably play a role in communicating group size and composition to conspecifics, and in finding partners to form new groups (Pollock 1986; Giacoma et al. 2010). Indri songs are essentially of three types (Pollock 1986). *Cohesion songs* are emitted when the group members are not in visual contact. *Territorial songs* are performed when two groups meet at the border of their territory and engage in singing battles. However, the most common song that indris daily emit is known as *advertisement song* whose main purpose is to signal their presence to conspecific groups nearby (Torti et al. 2013). Furthermore, indri songs adhere to the laws of compression as follows: they show a trade-off between the length of the signal and that of its components and a balance between signal duration and occurrence (Valente et al. 2021), suggesting the need for compensating the cost of sustained vocal emission.

This study aims at understanding whether vocal fatigue may represent a proxy for the occurrence of NLP within the song of the indris. To answer this question, we investigated whether the total occurrence of NLP may be explained by the duration of the vocal display and increase as singing proceeds. We also investigated whether NLP varies between seasons and occur evenly between the two sexes and across different age classes.

**Fig. 1:**
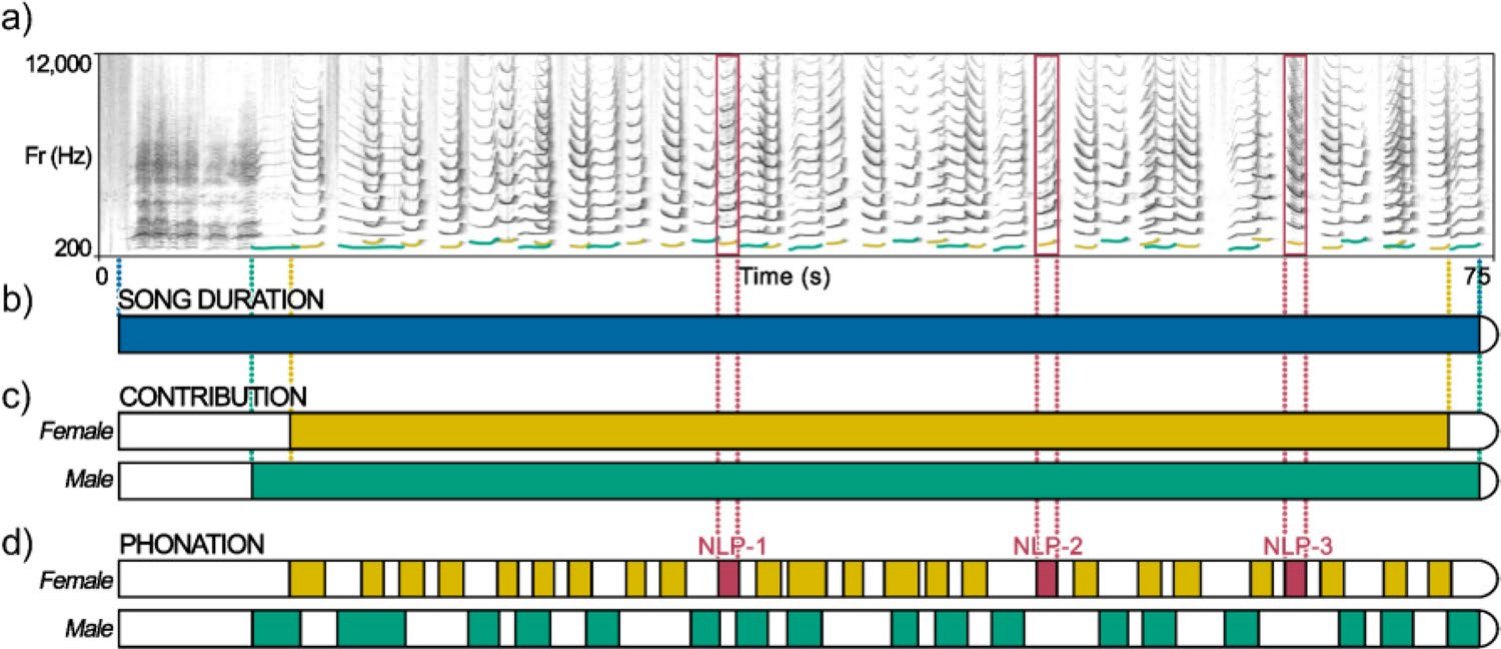
**a** Spectrogram of an *advertisement song* of an adult male and an adult female indri singing in the Maromizaha forest (Madagascar). The fundamental frequency is highlighted in ochre (female) and green (male). Red dotted lines highlight NLP. **b** Blue bar shows *song duration*, i.e., the duration between the beginning of the first roar to the end of the last unit of the duet or chorus. **c** An ochre and green bar show the female and male individual *contributions*, i.e., the duration between the beginning of the first unit of an individual and the end of the last unit of the same individual. **d** An ochre (female) and green (male) sections of the bars display the *phonation* of both sexes, i.e., the sum of the duration of all the units of a specific individual contribution. Among phonation intervals, red denotes the NLP, which are numbered consecutively

Since the presence of nonlinearities in animal vocalizations has been considered a potential indicator of vocal fatigue (Riede et al. 2004; Boucher 2008), we hypothesized an effect of the duration of the vocal display on the occurrence of NLP due to a trade-off between the need for conveying important information to conspecifics and the effort required to perform long, complex, and potentially exhausting vocal performances. To test this hypothesis, we considered the vocal display in terms of three temporal variables, namely the *song duration*, i.e., the length of the song, the duration of the individual *contribution*, i.e., the duration of the individual singing including silences between one uttered unit and another, and the duration of the individual *phonation*, i.e., the cumulative duration of all the uttered units (Fig. 1). We then tested which of these variables could better explain the total occurrence of NLP within a song. Considering that the individual phonation corresponds to the actual individual engagement and may, therefore, better reflect a short-term physical effort, we predicted that (1) this may be the best variable to explain the total occurrence of NLP within a song among the three temporal variables we considered. Furthermore, we tested whether NLP have a higher probability to be emitted at the beginning or at the end of the individual’s phonation. We predicted that (2) NLP distribution may change depending on the timing of the signal, i.e., the initial or the final portion of the song, and increase in number over time as the vocal display proceeds. We also hypothesized an effect of the season on the total NLP occurrence. We therefore predicted (3) a higher occurrence of nonlinearities during the reproductive season when indris sing more often (Ravaglia et al. 2023) and may be more prone to long-term vocal fatigue. Last, we hypothesized that NLP occurrence may be influenced by the sex and the age of the singer due to the different vocal behavior and engagement of adults and juveniles of both sexes to the song. Given that females emit a higher number of units and phrases than males (Giacoma et al. 2010; Zanoli et al. 2020), show longer contributions, and exhibit shorter intervals between the onsets of two consecutive units compared to males (De Gregorio et al. 2019), we predicted (4) that female singing may be more difficult to sustain and that, therefore, female contributions may show a higher NLP occurrence than male ones. We also predicted (5) that NLP may occur earlier in females who may suffer vocal fatigue before than males. Furthermore, since previous research showed that NLP may play a role in making signals unpredictable and preventing the habituation of listeners and that this may be particularly effective for those individuals who are potentially more exposed to dangers or need more attention (Fitch et al. 2002; Blumstein and Récapet 2009; Karp et al. 2014), we predicted that 6) juveniles exhibit a higher occurrence of NLP than adults.

## Methods

### Subjects and recordings

Between 2010 and 2020, we recorded the songs of seven habituated groups of wild indris living in the Maromizaha New Protected Area (18°56′49′′S, 48°27′53′′E, Madagascar). We selected 15 advertisement songs (Torti et al. 2013; Fig. 1a) for each group (duets and choruses). Out of the 105 songs, 64 were recorded during the reproductive season (September–February, Ravaglia et al. 2023), and 41 during the non-reproductive season (March-August, Ravaglia et al. 2023). We recorded the songs at a 2–10 m distance using solid-state recorders (sampling rate: 44.1 kHz; 16-bit resolution) connected to shotgun microphones oriented toward the emitters. All the songs were spontaneously emitted by the individuals, without the use of playback stimuli. By using focal animal sampling (Altmann 1974), we were able to discriminate between the singing individuals and attribute each vocalization to its emitter. See S1 *Supplementary Information* for details on the identity, group, sex, age, season, and number of contributions per individual who engaged in the songs we used in this study.

### Acoustic analysis

We used Praat 6.2.07 (Boersma and Weenink 2022) to visually inspect each song spectrogram. A first operator (WC) manually labeled all the vocal units of the singer and identified NLP (.Textgrid format; labels: linear or nonlinear). Examples of NLP are shown in S2 – *Supplementay Information*. We then randomly selected 10% of the contributions from our sample, and a second operator (TR) separately labeled the units following the same methodology. We reached an inter-rater agreement equal to 89.98% and a proportion of observed agreement of 0.88 out of all the labeled units. We then calculated the Cohen’s Kappa, taking into account the inter-observers chance agreement, obtaining a *K* = 0.72 (values between 0.40 and 0.75 are considered to represent a good agreement, values above 0.75 represent excellent agreement – Fleiss et al. 2003). We did not consider *roars* for our NLP count as their structure prevents the recognition of their actual emitter (Valente et al. 2021), although we included them when measuring the duration of each song. We measured three temporal variables (Fig. 1 b-d and S3 – *Supplementary Information*): *song duration*, from the beginning of the first *roar* to the ending of the last modulated unit; the duration of the individual contributions (*contribution*), from the beginning of the first modlated unit to the ending of the last modulated unit (long notes and descending phrases, see Giacoma et al. 2010) emitted by a singer; the individual phonation (*phonation*), i.e., the cumulative duration of all the units emitted within a *contribution* (De Gregorio et al. 2019). We calculated such temporal variables to be able to test our first two hypotheses. Since the songs were recorded at different distances and in different weather conditions, we were not able to reliably assess a measure of the loudness. However, previous data revealed that indri songs can reach 110 dB at 0.50 m (Zanoli et al. 2020), and there is no evidence that the loudness of the song varies according to the duration. We, therefore, were not able to test whether and how much loudness played a role in the NLP occurrence.

### Statistical analysis

We built three Generalized Linear Mixed Models (GLMM; *glmmTMB* package, R Core Team 2021, version 4.1.2), each testing for the effect of one temporal variable (*song duration*, *contribution*, or *phonation;* predictors) on the occurrence of NLP. The response variable followed a Poisson distribution, as specified in each model. W e also tested the effect of sex (female or male) and age (i.e., adults, from 6 years old and juveniles, i.e., non-reproductive individuals, from 2 years old, Rolle et al. 2021) in interaction, and season (reproductive or non-reproductive). Each model included a song code and an individual code, i.e., unique acronyms indicating a particular song and a particular individual as random factors. With the aim of understanding which of the three temporal variables better explained the occurrence of NLP in songs, we selected the model showing the best goodness of fit first through a likelihood ratio test (Anova with argument test “Chisq”; Dobson 2002) to compare each model with one another, then by using Efron’s pseudo R-squared test (*performance* package).

We then split the temporal variable contained in the best-fitting model into duration quartiles. We counted the number of NLP occurring in each quartile to understand if the NLP distribution changed as vocal display proceeded. To test this hypothesis, we ran a GLMM using NLP occurrence within each quartile as the response variable (one count of NLP occurrence per every quartile), which followed a Poisson distribution. The explanatory variables were the temporal variable, its quartiles (as an integer variable), the interaction between sex and age, and season. The model included the song and individual identity codes as random factors.

For all the models we ran, we verified the significance of the full model (including explanatory variables and random factors) versus the null model (including random factors only) through a likelihood ratio test (Anova with argument test “Chisq”, Dobson 2002). We also verified the assumptions that the residuals were normally distributed and homogenous by looking at a *qqplot* and the distribution of the residuals plotted against the fitted values (a function provided by R. Mundry). We excluded the presence of collinearity among predictors by verifying the Variance Inflation Factors (VIFs, *performance* package). We also performed pairwise comparisons for each level of the factors in the model using a post-hoc test (*emmeans* package). Finally, potential overdispersion was evaluated for each model using *performance* package in R. None of the models was flawed by overdispersion.

To test whether NLP occur earlier in females than males, we also tested whether the timing of the onset of NLP differed among sex and age classes by looking at how quickly the first nonlinear phenomenon occurs, i.e., latency to first nonlinear phenomenon. To do so, we calculated the time the first nonlinear phenomenon occurred in an individual contribution and performed a survival analysis in R (*ggsurvFit* and *survival* packages). We first generated Kaplan–Meier plots to show the survival curves, i.e., time-to-event endpoints by using the time at which the first nonlinear phenomenon occurred as the response variable and the interaction between sex and age as the predictor variable, and we calculated the average survival time, i.e., the average latency to first nonlinear phenomenon for each predictor level, i.e., females, males, adults, and juveniles. We then compared the survival times between groups by using a log-rank test. Finally, we performed pairwise comparisons for each factor level in the model by using a post-hoc (*survminer* package).

## Results

**Fig. 2:**
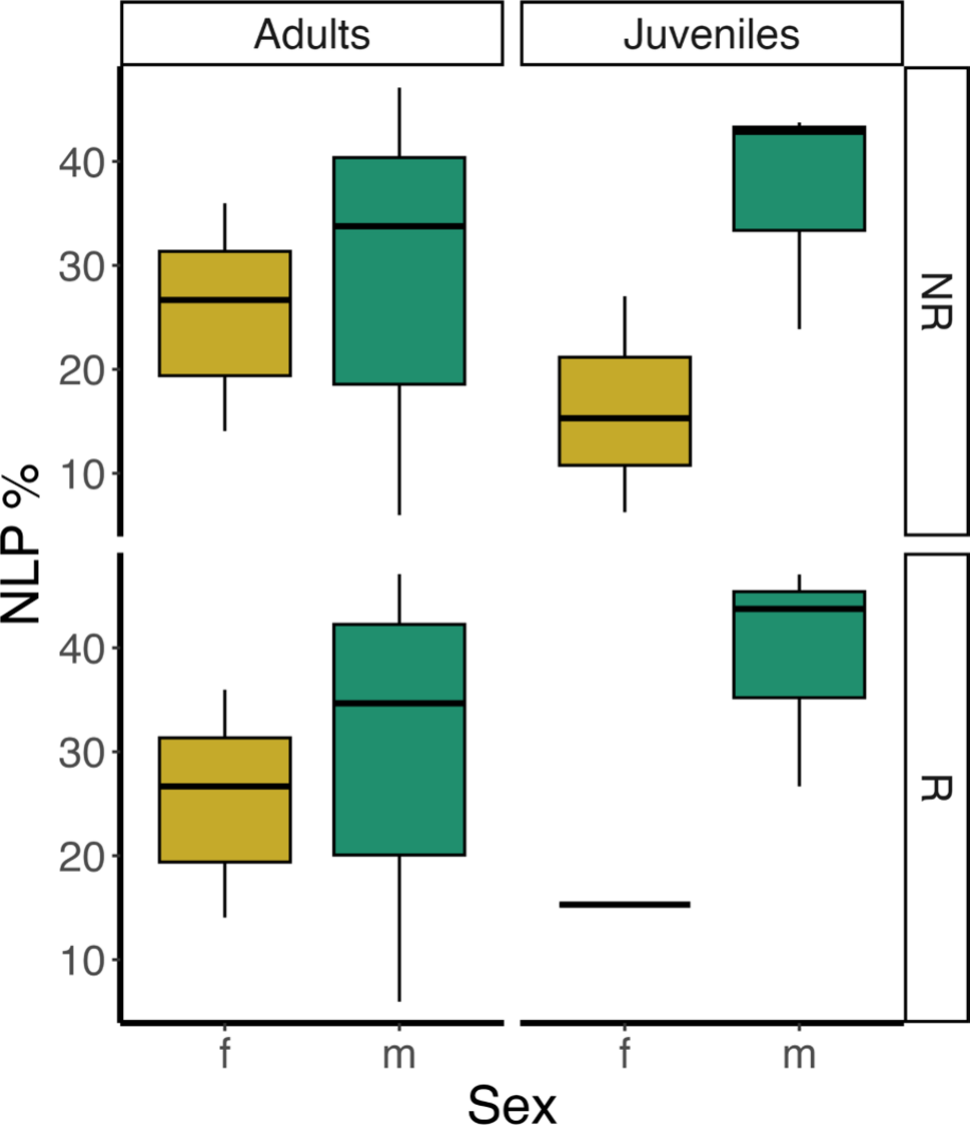
Percentage (%) of NLP in the song units given by the 28 sampled individuals of indris (total n of emitted units = 4995; total n of NLP = 1418). The percentage is calculated by dividing the number of units showing NLP for a particular individual by the total number of emitted notes. We show the results by sex (female, male), age (adult and juvenile) and season (non-reproductive, reproductive)

**Fig. 3:**
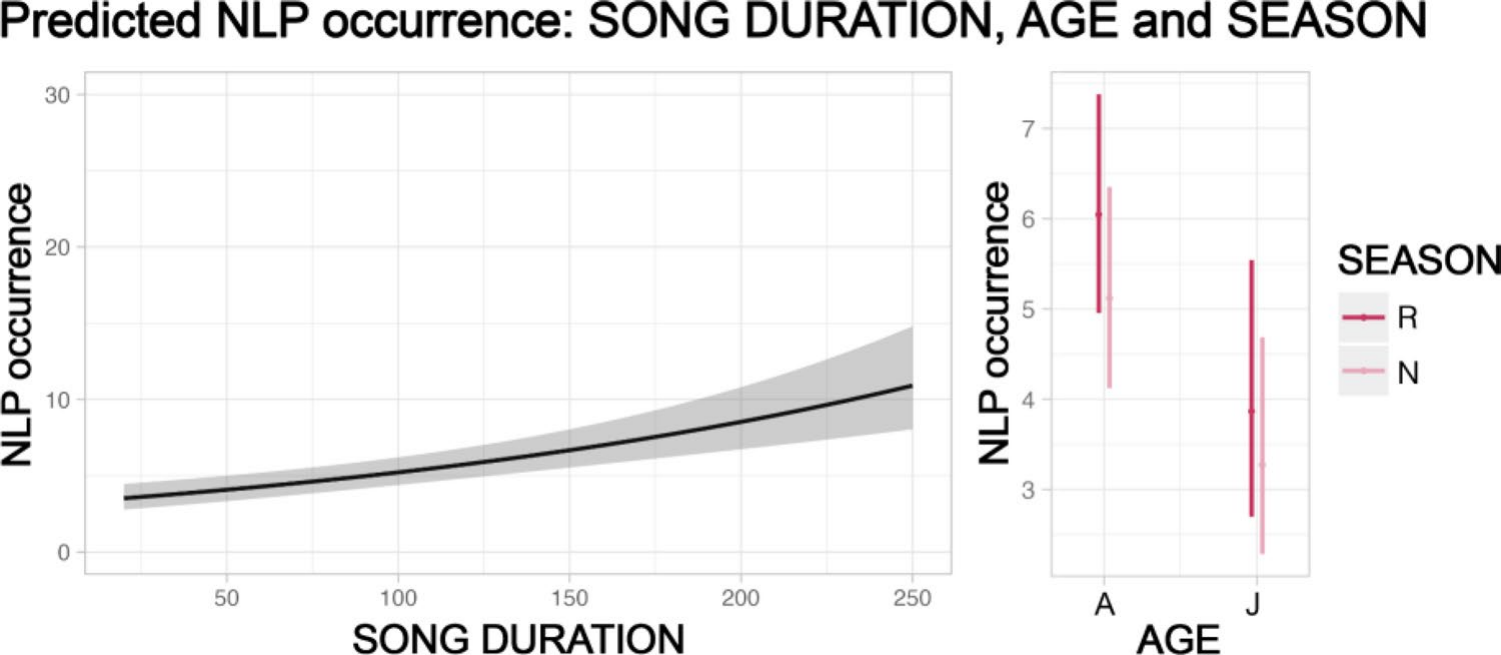
Effect plots of the GLMM, considering sex and age and their interaction, testing for the effect of the temporal variable song duration (in seconds) on the occurrence of NLP (shaded areas indicate 95% confidence intervals; observations *n* = 227). Song duration positively and significantly affects the occurrence of NLP. Age (A = adult, J = juvenile) and season (NR = non-reproductive, R = reproductive) have a significant effect on the occurrence of NLP, and are displayed on the right with effect plots (boxplots). Sex alone (F = female, M = male) and the interaction between sex and age were not significant and therefore not shown

**Fig. 4:**
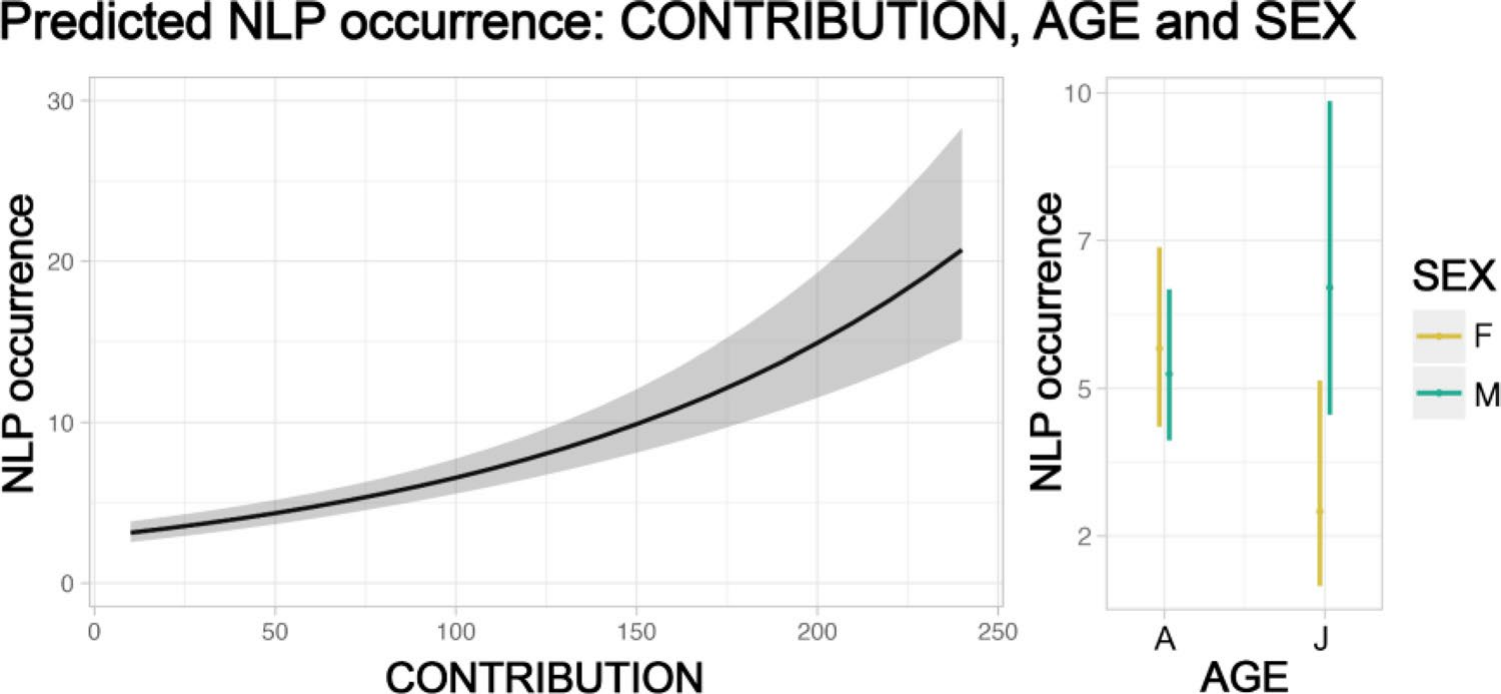
Effect plots of the GLMM, considering sex and age and their interaction, testing for the effect of the temporal variable contribution (in seconds) on the occurrence of NLP (shaded areas indicate 95% confidence intervals; observations *n* = 227). Contribution positively and significantly affects the occurrence of NLP. Age (A = adult, J = juvenile) and the interaction between age and sex (F = female, M = male) have a significant effect on the occurrence of NLP and are displayed on the right with effect plots (boxplots). Season does not have an effect on NLP occurrence and therefore not shown

**Fig. 5:**
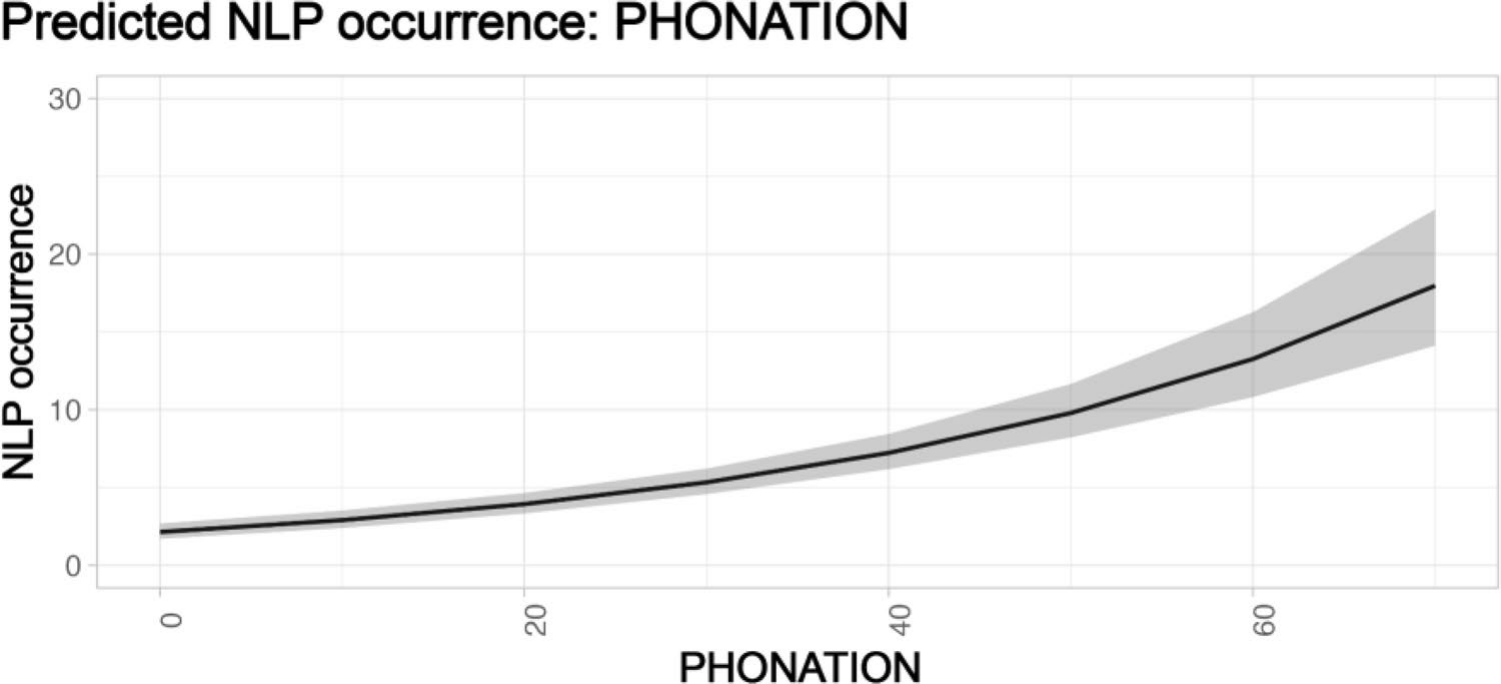
Effect plots of the GLMM, considering sex and age and their interaction, testing for the effect of the temporal variable phonation (in seconds) on the occurrence of NLP (shaded areas indicate 95% confidence intervals; observations *n* = 227). Phonation positively and significantly affects the occurrence of NLP. No factors among age (A = adult, J = juvenile), sex (F = female, M = male), their interaction or season (R = reproductive, NR = non-reproductive) have a significant effect on the occurrence of NLP and therefore not shown

In 245 contributions emitted by 28 individuals (8 adult females, 11 adult males, 3 juvenile females, 5 juvenile males, and 1 female whose age was unknown), we identified 1418 NLP (618 emitted by adult females, 642 by adult males, 25 by juvenile females, and 133 by juvenile males). 952 out of 1418 NLP were emitted during the reproductive season. Contributions per sex and age were distributed as follows: 99 for adult females, 110 for adult males, 11 for juvenile females, 23 for juvenile males, and 2 for a female whose age was unknown and was, therefore, not included in the analyses. The percentage of NLP on the total number of emitted units per sex, age, and season is shown in Fig. 2. For each of the three models testing the effect of temporal variables on total NLP occurrence, the three *full* models were significantly different from the *null* ones (*song duration*: Chisq = 35.549, df = 8, *p* < 0.001; *contribution*: Chisq = 85.556, df = 8, *p* < 0.001; *phonation*: Chisq = 118.421, df = 8, *p* < 0.001). Furthermore, for all models, the temporal variables had a significant effect on the number of NLP (Figs. 3, 4, 5; *song duration*: estimate = 0.005, *z* = 5.343, *p* < 0.001; *contribution*: estimate = 0.008, *z* = 9.480, *p* < 0.001; *phonation*: estimate = 0.030, *z* = 11.680, *p* < 0.001; Tables 1, 2 ,3).

**Table 1:** Effects of the predictors (*song duration*, ex, age, season, and the interaction between sex and age) on the total occurrence of NLP; results of the full model and post-hoc comparisons

Generalized Linear Mixed Model (GLMM) Family = Poisson Count (NLP occurrence) ~ *Song duration* + Sex*Age + Season + (1|song ID) + (1|individual ID) Full vs Null (chisq = 34.973, df = 8, *P* < 0.001)				
	Estimate	SE	*z* value	*P*
(Intercept)	1.372	0.175	^a^	^a^
*Song duration*	0.005	0.000	5.343	** < 0.001**
Sex (M)^b,c^	– 0.154	0.188	– 0.822	0.411
Age (J)^b,c^	– 0.877	0.329	– 2.670	**0.008**
Season (NR)^b,d^	– 0.167	0.084	– 1.976	**0.048**
Sex*Age (M, J)^b,c^	0.794	0.406	1.955	0.051

Post-hoc comparisons						
Emmeans						
Sex	Age	Emmean	SE	df	lower.CL	upper.CL
F	A	1.800	0.141	219	1.521	2.08
M	A	1.645	0.129	219	1.391	1.90
F	J	0.923	0.298	219	0.335	1.51
M	J	1.562	0.207	219	1.155	1.97

Contrasts					
Contrast	Estimate	SE	df	t.ratio	*P*
FA – MA	0.154	0.188	219	0.822	0.844
FA – FJ	0.877	0.329	219	2.670	**0.040**
FA – MJ	0.237	0.247	219	0.960	0.772
MA – FJ	0.723	0.322	219	2.247	0.114
MA – MJ	0.083	0.240	219	0.346	0.986
FJ – MJ	– 0.640	0.359	219	– 1.782	0.285

Factor levels sex = ‘male’ (M) and ‘female’ (F); factor levels age = ‘juvenile’ (J) and ‘adult’ (A); factor levels season = ‘non-reproductive’ (NR) and ‘reproductive’ (R)

Bold was used to highlight the statistically significant *P*-values

^a^Not shown as not having a meaningful interpretation

^b^Estimate ± SE refer to the difference of the response between the reported level of this categorical predictor and the reference category of the same predictor

^c^These predictors were dummy coded, with the “Sex (Female)”, and “Age (Adult)” being the reference categories

^d^This predictor was dummy coded, with the “Season (Reproductive)” being the reference category

**Table 2:** Effects of the predictors (individual *contribution*, sex, age, season, and the interaction between sex and age) on the total occurrence of NLP; results of the full model and post-hoc comparisons

Generalized Linear Mixed Model (GLMM) Family = Poisson Count (NLP occurrence) ~ *Contribution* + Sex*Age + Season + (1|song ID) + (1|individual ID) Full vs Null (chisq = 164.723, df = 8, *P* < 0.001)				
	Estimate	SE	*z* value	*P*
(Intercept)	1.147	0.161	^a^	^a^
*Contribution*	0.008	0.000	9.480	** < 0.001**
Sex (M)^b,c^	– 0.078	0.180	– 0.433	0.665
Age (J)^b,c^	– 0.665	0.318	– 2.090	**0.037**
Season (NR)^b,d^	– 0.048	0.077	– 0.620	0.535
Sex*Age (M, J)^b,c^	0.911	0.394	2.312	**0.021**

Post-hoc comparisons						
Emmeans						
Sex	Age	Emmean	SE	df	lower.CL	upper.CL
F	A	1.73	0.135	219	1.464	2.00
M	A	1.65	0.123	219	1.409	1.90
F	J	1.07	0.289	219	0.496	1.63
M	J	1.90	0.197	219	1.510	2.29

Contrasts					
Contrast	Estimate	SE	df	t.ratio	*P*
FA – MA	0.078	0.180	219	0.433	0.973
FA – FJ	0.665	0.318	219	2.090	0.159
FA – MJ	– 0.167	0.238	219	– 0.703	0.896
MA – FJ	0.587	0.312	219	1.881	0.239
MA – MJ	– 0.245	0.232	219	– 1.057	0.716
FJ – MJ	– 0.833	0.349	219	– 2.386	0.083

Factor levels sex = ‘male’ (M) and ‘female’ (F); factor levels age = ‘juvenile’ (J) and ‘adult’ (A); factor levels season = ‘non-reproductive’ (NR) and ‘reproductive’ (R)

Bold was used to highlight the statistically significant *P*-values

^a^Not shown as not having a meaningful interpretation

^b^Estimate ± SE refer to the difference of the response between the reported level of this categorical predictor and the reference category of the same predictor

^c^These predictors were dummy coded, with the “Sex (Female)”, and “Age (Adult)” being the reference categories

^d^This predictor was dummy coded, with the “Season (Reproductive)” being the reference category

**Table 3:** Effects of the predictors (individual *phonation*, sex, age, season, and the interaction between sex and age) on the total occurrence of NLP; results of the full model

Generalized Linear Mixed Model (GLMM) Family = Poisson Count (NLP occurrence) ~ *Phonation* + Sex*Age + Season + (1|song ID) + (1|individual ID) Full vs Null (chisq = 238.944, df = 8, *P* < 0.001)				
	Estimate	SE	*z* value	*P*
(Intercept)	0.861	0.161	^a^	^a^
*Phonation*	0.030	0.003	11.680	** < 0.001**
Sex (M)^b,c^	– 0.156	0.171	– 0.917	0.359
Age (J)^b,c^	– 0.480	0.307	– 1.565	0.118
Season (NR)^b,d^	0.018	0.070	0.260	0.795
Sex*Age (M, J)^b,c^	0.604	0.377	1.604	0.109

Factor levels sex = ‘male’ (M) and ‘female’ (F); factor levels age = ‘juvenile’ (J) and ‘adult’ (A); factor levels season = ‘non-reproductive’ (NR) and ‘reproductive’ (R)

Bold was used to highlight the statistically significant *P*-value

^a^Not shown as not having a meaningful interpretation

^b^Estimate ± SE refer to the difference of the response between the reported level of this categorical predictor and the reference category of the same predictor

^c^These predictors were dummy coded, with the “Sex (Female)”, and “Age (Adult)” being the reference categories

^d^This predictor was dummy coded, with the “Season (Reproductive)” being the reference category

Considering the effect of season, sex, and age, the model with *song duration* as a predictor showed a significant effect of age and season on NLP occurrence (age: estimate =  – 0.877, *z* =  – 2.670, *p* = 0.008; season: estimate = 0.167, *z* =  – 1.976, *p* = 0.048; Table 1). Specifically, the number of NLP was significantly higher in adult than in juvenile females and in the reproductive than in the non-reproductive season (Table 1).

The model with *contribution* as a predictor showed a significant effect of both age and its interaction with sex overall (age: estimate =  – 0.665, *z* =  – 2.090, *p* = 0.037; sex*age: estimate = 0.911, *z* = 2.312, *p* = 0.021; Table 2), but no significant post-hoc comparisons (Table 2). The number of NLP was significantly higher in adults than juveniles. Moreover, age had a significant effect on NLP occurrence also in interaction with sex, although no contrasts significantly differed from one another (Table 2).

The model including *phonation* as a predictor did not show any significant effect other than phonation and no significant post-hoc contrasts, i.e., age, sex, interaction, and season did not predict the occurrence of NLP (Table 3).

When comparing the three models, we found that the model including *phonation* as a predictor for NLP occurrence was the best-fitting one (pseudo R^2^ *song duration*: 0.632; pseudo R^2^ *contribution*: 0.645; pseudo R^2^ *phonation*: 0.652; so*ng duration* vs *contribution*: Chisq = 50.007, df = 8, *p* < 0.001; *contribution* vs *phonation*: Chisq = 35.865, df = 8, *p* < 0.001; *song duration* vs *phonation*: Chisq = 82.872, df = 8, *p* < 0.001).

**Fig. 6:**
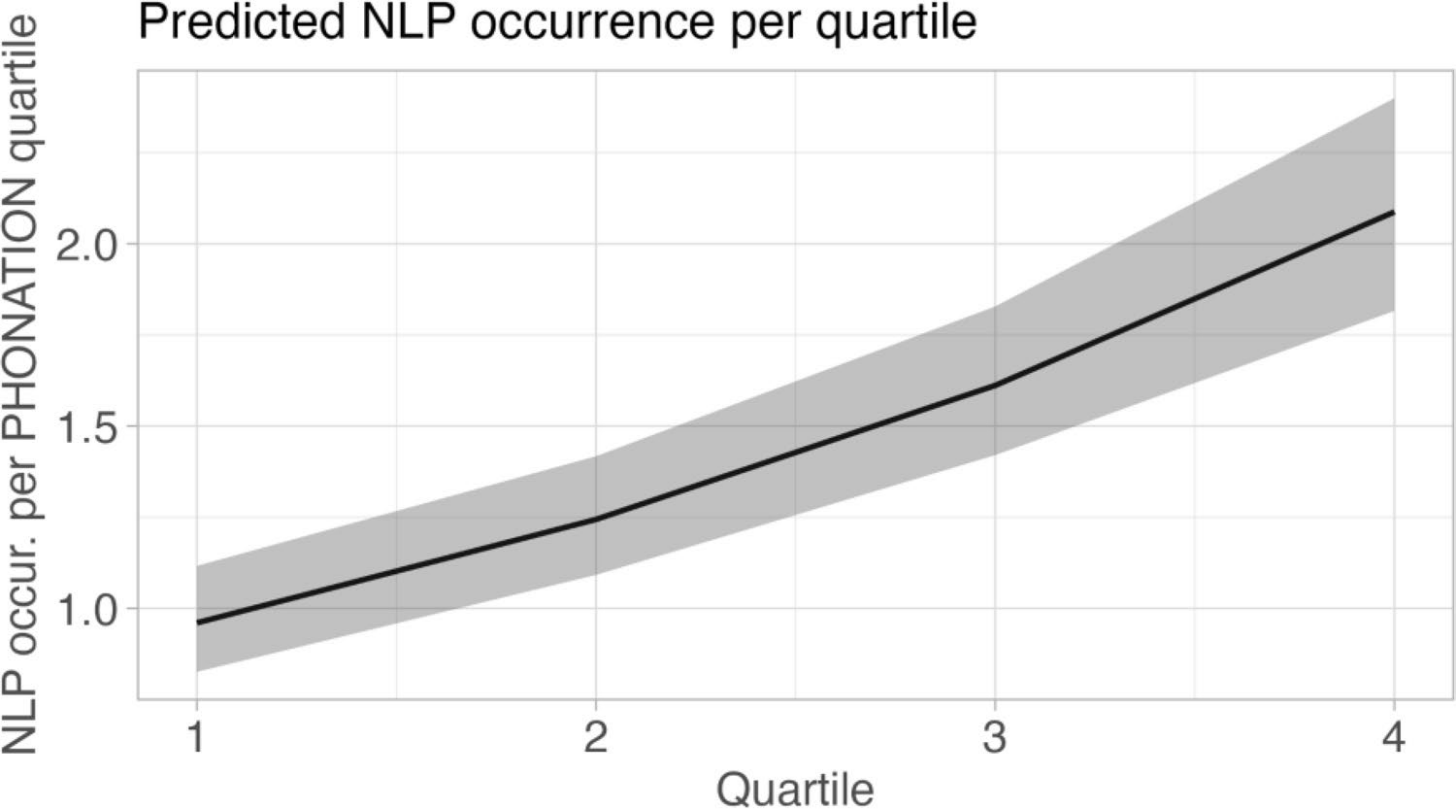
Effect plot of the GLMM testing for the effect of the quartile of phonation, *phonation*, sex, age, and their interaction on the occurrence of NLP in the specific quartile. The model showed that the occurrence of NLP increased with *phonation* and quartile. Shaded areas indicate 95% confidence intervals

Considering that, we split *phonation* into duration quartiles and found that the occurrence of NLP increased with quartiles Fig. 6; *phonation*: estimate = 0.028, *z* = 12.200, *p* < 0.001; quartiles: estimate = 0.259, z = 10.598, *p* < 0.001; Table 4). We did not detect a significant effect of sex, age, their interaction, and season on NLP occurrence (Table 4).

**Table 4:** Effects of the predictors (individual *phonation*, phonation quartiles, sex, age, season, the interaction between sex and age, and the interaction between individual *phonation* and quartiles) on the occurrence of NLP within the quartiles of phonation; results of the full model

Generalized Linear Mixed Model (GLMM) Family = Poisson Count (NLP within the quartiles of phonation) ~ *Phonation* + Quartiles + Sex*Age + Season + (1|song ID) + (1|individual ID) Full vs Null (chisq = 247.546, df = 10, *P* < 0.001)				
	Estimate	SE	*z* value	*P*
(Intercept)	– 1.069	0.152	^a^	^a^
*Phonation*	0.028	0.002	12.200	** < 0.001**
Quartiles	0.259	0.024	10.598	** < 0.001**
Sex (M)^b,c^	– 0.142	0.140	– 1.016	0.310
Age (J)^b,c^	– 0.480	0.272	– 1.764	0.078
Season (NR)^b,d^	0.046	0.064	0.712	0.476
Sex*Age (M, J)^b,c^	0.518	0.328	1.580	0.114

Bold was used to highlight the statistically significant *P*-values

^a^Not shown as not having a meaningful interpretation

^b^Estimate ± SE refer to the difference of the response between the reported level of this categorical predictor and the reference category of the same predictor

^c^These predictors were dummy coded, with the “Sex (Female)”, and “Age (Adult)” being the reference categories

^d^This predictor was dummy coded, with the “Season (Reproductive)” being the reference category

**Fig. 7:**
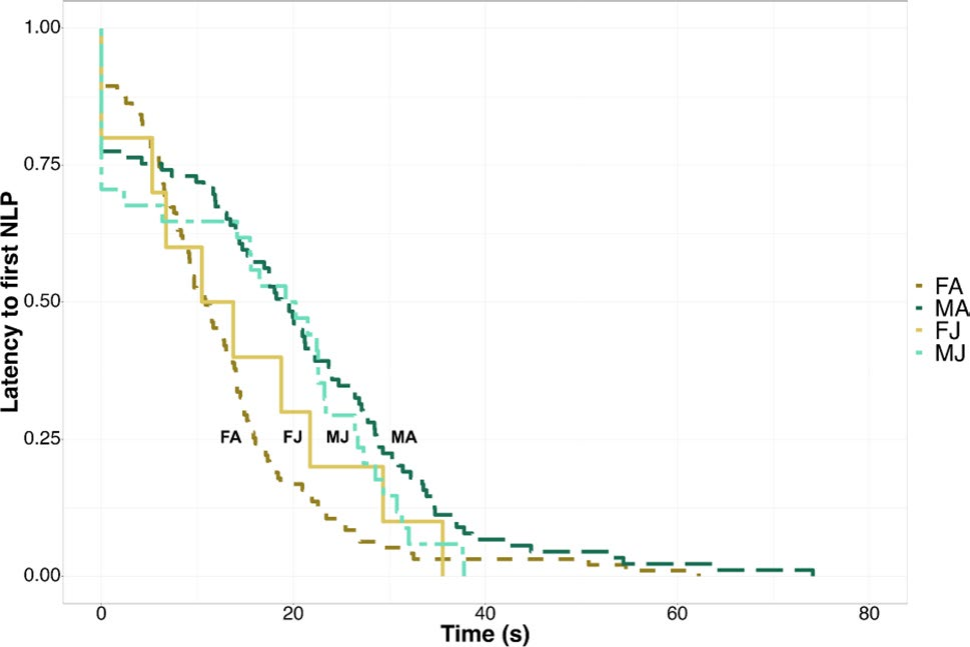
Kaplan–Meier curves plotting the time at which the first nonlinear phenomenon occurred within an individual contribution (x-axis) and the probability of emitting the first nonlinear phenomenon after a certain period of time (y-axis). Factor levels sex = ‘male’ (M) and ‘female’ (F); factor levels age = ‘juvenile’ (J) and ‘adult’ (A)

Kaplan–Meier plots show the survival curves for all the four categories we considered in the survival analysis, i.e., adult females, adult males, juvenile females, and juvenile males (Fig. 7). We found that there was a significant difference in the survival curves and, therefore, in the latency to the first nonlinear phenomenon between sex and age classes as resulted from the log-rank test (Chisq = 17.700, df = 3, *p* < 0.001; S4 – *Supplementary Information*). Specifically, half of the time adult females emitted their first nonlinear phenomenon within 10.848 s (average survival time = 10.848 s), whereas half of the time the first nonlinear phenomenon for adult males occurred within 18.784 s (average survival time = 18.784 s). The post-hoc revealed that this difference was significant, showing that, on average, the first nonlinear phenomenon occurs earlier in adult females than adult males (*p* < 0.001, S4—*Supplementary Information*).

## Discussion

Our study aimed to investigate whether vocal fatigue may play a role in the emission of NLP and whether season, sex, and age may influence such phenomena accordingly. We demonstrated that NLP occurrence is best predicted by the *phonation*, although also *song duration* and *contribution* showed a positive effect on the response variable. Furthermore, NLP progressively increase with *phonation*. We also searched for an effect of the seasonality, and we found that for the same duration of the song, the number of NLP was smaller during the non-reproductive season. Moreover, we tested whether sex affects the occurrence of NLP, and although we did not identify sex differences in terms of total occurrence, we found that adult females emit their first nonlinear phenomenon earlier than adult males. Finally, we found that being an adult is associated with increased NLP within *song duration* and *contribution*, and that adult females produce significantly more NLP than juvenile females within the song.

We provided evidence for a positive relationship between the duration of the vocal display and NLP occurrence, and we showed that *phonation* is the best predictor among the three temporal variables we considered. We also found a positive effect of the phonation quartiles, indicating that NLP increase over time and are likely to occur more often during the final stages of the *phonation*. Our results confirm our first two predictions and suggest that effective engagement in singing determines how many NLP an individual emits. Like in chimpanzees’ vocalizations (Riede et al. 2004, 2007) and hyrax songs (Weissmann et al. 2020), intense vocal activity may favor the emergence of vocal nonlinearities. Indeed, the production of harmonic sounds may be limited by physiological constraints while singing long and demanding calls; as such, indris may experience vocal fatigue leading to an increase in NLP production. In agreement with previous studies on humans (Boucher 2008) and chimpanzees (Riede et al. 2004), vocal fatigue may thus foster the deterioration of vocal emission.

Although we predicted that NLP occurrence may be significantly higher during the reproductive season, when indris sing more often, we only found a weak positive effect of the season in the first model we built. NLP occurrence shows a low seasonal pattern related to the *song duration*, but we did not detect any effect concerning either the *contribution* or *phonation*. This finding may suggest that a certain degree of fatigue might be experienced by indris every time they emit songs, regardless of the season. According to our results, whether there is an accumulation of fatigue in indris during particular periods of the year is unclear, in contrast with previous findings that reported a change in NLP rates in the male calls of fallow bucks (*Dama dama*) according to a seasonal pattern (Vannoni and McElligott 2009) or demonstrated how the prolonged effort needed to sustain long calls leads to vocal fatigue (Castellano and Gamba 2011).

Considering sex and age in interaction, we found an overall effect of these two terms in the model including *contribution* as a predictor. However, we found no support for our prediction that NLP occurrence is higher in females than males, in disagreement with previous findings on baboons’ loud calls (Fischer et al. 2004), humpback whales’ songs (Cazau et al. 2016), and rock hyraxes’ songs (Demartsev et al. 2016). We found sex differences only in terms of latency to the first nonlinear phenomenon. Indeed, based on our findings, adult females are likely to emit their first nonlinear phenomenon earlier than adult males, i.e., there is a significant difference in the latency to the first nonlinear phenomenon between adult females and males, in agreement with what we predicted. Therefore, our results show that despite both sexes experiencing similar levels of fatigue, adult females could be affected by the vocal effort quicker than adult males, given that females emit more units and more phrases (Giacoma et al. 2010; Zanoli et al. 2020) and take shorter intervals in between units compared to males (De Gregorio et al. 2019). Hence, adult males may be able to rest and maintain vocal control more easily yet we cannot exclude that any difference in the way indris emit the first nonlinear phenomenon might be also related to sex-specific factors, such as female hormone levels during oestrus, which might affect the vocal behavior and sound production, as previously underlined in humans (Raj et al. 2010) and giant pandas (Charlton et al. 2018).

We finally rejected our prediction that juveniles emit NLP more frequently than adults. Instead, we found evidence that NLP occurrence is predicted by age but that adults are likely to emit more NLP than juveniles. This tendency is particularly strong between adult and juvenile females. Specifically, with an equal duration of the song, adult females tend to produce more NLP than juvenile ones. However, the small sample size of the juvenile females might partially affect this result. Our findings disagree with the hypothesis that NLP may be produced more by younger individuals to make their signals harder to ignore (Fitch et al. 2002; Massenet et al. 2022). However, a higher NLP occurrence in adults may suggest a loss in larynx control in older individuals, representing an aging indicator similar to what has been reported in dogs (Marx et al. 2021).

Whether physiological constraints hamper the emission of harmonic units, NLP may result as a mere byproduct of energetically expensive vocalizations (Zanoli et al. 2020) or a specific phonatory system (Gamba et al. 2011). This might be due to a compensatory mechanism balancing the cost of sustaining long calls. It has been observed that NLP may be typical of species that exhibit peculiar communication systems basend on exhausting and loud calls, such as anurans (Feng et al. 2009) and whales (Cazau et al. 2016). Although essentially depending on physiological constraints emerging when performing long calls, NLP emission might be still improving unpredictability in the indris’ songs, thus facilitating other groups’ reception of their signals at long distances in the dense rainforest they inhabit. In this sense, NLP could have cognitive implications for a receiver. Indeed, where advertisement songs take the shape of long modulated sequences, neurally cheap unpredictability may serve the adaptive functions of making nonlinear vocalizations challenging to ignore, similar to rhesus macaque calls (Fitch et al. 2002). Even if those songs are critical to understanding the position in the home range of particular groups and space animals in the different forest patches (Torti et al. 2013, 2017), they can ignore those howling sounds while, for instance, continuing feeding on a particular tree. In this framework, cognitive processes may guide perceptual dynamics by classifying calls based on experience, potentially attributing the presence of NLP to a greater or lesser degree of urgency of the received signal.

In conclusion, despite NLP being common in the vocal repertoire of non-human primates (Fitch et al. 2002; Nishimura et al. 2022), the study of these features is still in its infancy. Our work investigated and quantified the occurrence of NLP in the songs of a lemur species. Addressing this topic in other primate species will be extremely important in framing the occurrence of NLP among the Strepsirrhini sub-order and among singing primates. Further studies will deepen our understanding of the occurrence of NLP across these *taxa* and provide new insights into the ecological role of these signals. For example, a more complete comprehension of the NLP occurrence will include differentiating and quantifying nonlinearities based on their spectral features.

## Supplementary Information

Below is the link to the electronic supplementary material.

Supplementary file1 (DOCX 282 KB)

## Data Availability

Data used in this study are available on request to the corresponding author and will be loaded on github once the manuscript will be accepted for publication.
