# Supplementary material for: Singing more, singing harsher: occurrence of nonlinear phenomena in a primate’ song
Source: Anim Cogn. 2023 Jul 17;26(5):1661–73. doi: 10.1007/s10071-023-01809-7 (PMC10442282; doi:10.1007/s10071-023-01809-7)
Supplement: Supplementary file 1 — Supplementary file1 (DOCX 282 KB) [file 10071_2023_1809_MOESM1_ESM.docx]

### SUPPLEMENTARY INFORMATION

**S1 - Supplementary methods - *Subjects and recordings***

Individual characteristics of the emitter and related number of contributions we considered in this study.

| **ID** | **Group** | **Sex** | **Age** | **Total contributions** | **Contributions in reproductive season** | **Contributions in non-reproductive season** |
| --- | --- | --- | --- | --- | --- | --- |
| Allblack | 5MZ | M | Juvenile | 1 | 1 | 0 |
| Befotsy | 6MZ | F | Adult | 15 | 8 | 7 |
| Bemasoandro | 8MZ | F | Adult | 15 | 3 | 12 |
| Bevolo | 1MZ | F | Adult | 14 | 12 | 2 |
| Cesare | 8MZ | M | Adult | 5 | 1 | 4 |
| Eme | 8MZ | M | Juvenile | 2 | 2 | 0 |
| Eva | 4MZ | F | Adult | 11 | 9 | 2 |
| Faly | 3MZ | M | Juvenile | 11 | 9 | 2 |
| Fern | 5MZ | F | Adult | 6 | 2 | 4 |
| Gibet | 4MZ | M | Adult | 4 | 3 | 1 |
| Graham | 5MZ | M | Adult | 15 | 6 | 9 |
| Hendry | 4MZ | M | Adult | 4 | 3 | 1 |
| Jery | 1MZ | M | Adult | 15 | 13 | 2 |
| Jonah | 8MZ | M | Adult | 12 | 3 | 9 |
| Kami | 1MZ | F | Juvenile | 2 | 0 | 2 |
| Koto | 4MZ | M | Adult | 11 | 8 | 3 |
| Mahagaga | 3MZ | M | Adult | 13 | 11 | 0 |
| Max | 2MZ | M | Adult | 15 | 9 | 6 |
| Mena | 3MZ | F | Adult | 14 | 12 | 2 |
| Meva | 4MZ | F | Juvenile | 6 | 4 | 2 |
| Mika | 8MZ | F | Juvenile | 3 | 0 | 3 |
| Ratsy | 3MZ | M | Adult | 1 | 1 | 0 |
| Soa | 2MZ | F | Adult | 15 | 9 | 6 |
| Takona | 5MZ | F | Adult | 9 | 4 | 5 |
| Tonga | 3MZ | F | *NA* | 2 | 2 | 0 |
| Voary | 5MZ | M | Juvenile | 3 | 0 | 3 |
| Zafy | 8MZ | M | Juvenile | 6 | 0 | 6 |
| Zokibe | 6MZ | M | Adult | 15 | 8 | 7 |

**S2 - Supplementary methods – *Acoustic analysis***

Examples of non-linear phenomena (NLP) occurring in the harmonic song of the indri. a) Male mixed note generated by a partial aperiodic oscillation of the vocal fold starting as a chaotic *roar* and ending as a harmonic unit. b) Differential vibrations of the vocal folds result in additional spectral bands (subharmonics) standing between the natural harmonics of a male unit. c) Amplitude modulation in a female unit resulting in additional spectral bands up and down the natural harmonics.

**
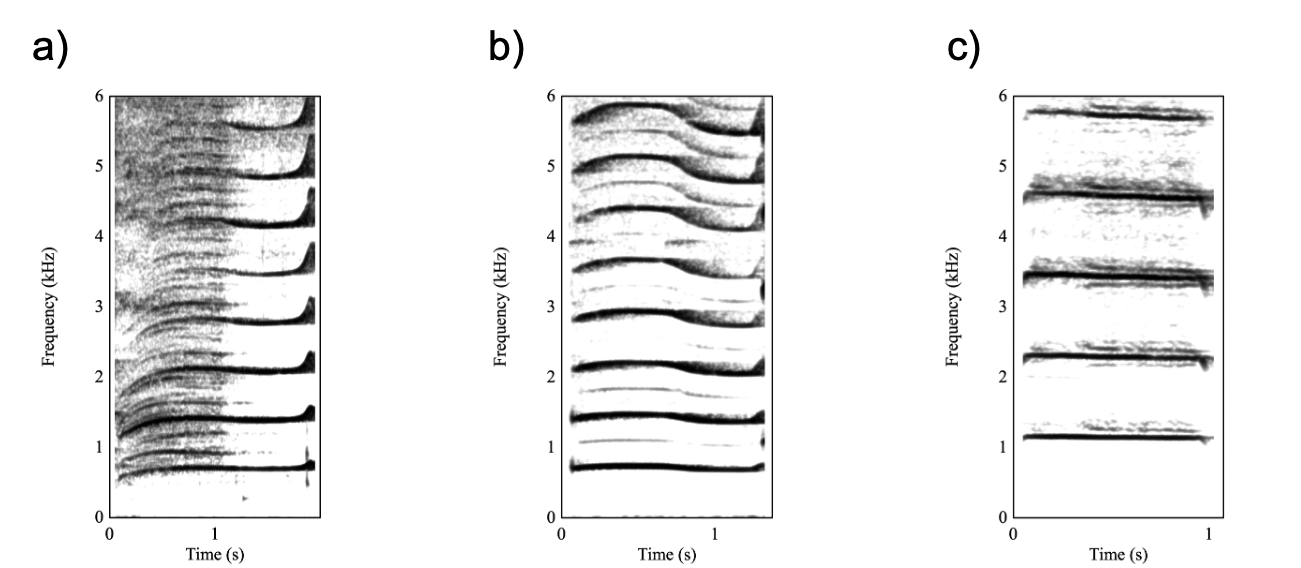
**

**S3 - Supplementary methods – *Acoustic analysis***

Summary statistics of the three temporal variables extracted from the songs and of the phonation quartiles we used as predictor. All the values are expressed in seconds (*s*).

| **Parameter** | **Min** | **Max** | **Mean** | **SD** |
| --- | --- | --- | --- | --- |
| ***Song duration*** | 22.750 | 244.900 | 104.900 | 47.301 |
| ***Contribution*** | 12.810 | 238.490 | 74.710 | 36.334 |
| ***Phonation*** | 6.968 | 70.508 | 30.125 | 11.736 |
| ***Phonation* quartiles** | 1.742 | 17.627 | 7.533 | 2.941 |

**S4 - Supplementary methods – *Results***

Log-rank test results and posthoc comparisons.

| **Log-rank test**  **Time of the first uttered nonlinear phenomenon ~ Sex*Age**  Full vs Null (chisq= 17.700, *df* = 3, *P* < 0.001) | | | | | |
| --- | --- | --- | --- | --- | --- |
|  | **N** | **Observed** | **Expected** | **(O-E)^2/E** | **(O-E)^2/V** |
| Adult females | 95 | 95 | 68.66 | 10.101 | 15.956 |
| Adult males | 89 | 89 | 114.56 | 5.701 | 12.434 |
| Juvenile females | 10 | 10 | 8.85 | 0.150 | 0.163 |
| Juvenile males | 34 | 34 | 35.93 | 0.104 | 0.130 |

| **Pairwise comparisons using log-rank test** | | | |
| --- | --- | --- | --- |
|  | Adult females | Adult males | Juvenile females |
| Adult males | **< 0.001** | - | - |
| Juvenile females | 0.508 | 0.357 | - |
| Juvenile males | 0.103 | 0.357 | 0.508 |
